# The Transcriptional Landscape of Berry Skin in Red and White PIWI (“Pilzwiderstandsfähig”) Grapevines Possessing QTLs for Partial Resistance to Downy and Powdery Mildews

**DOI:** 10.3390/plants13182574

**Published:** 2024-09-13

**Authors:** Francesco Scariolo, Giovanni Gabelli, Gabriele Magon, Fabio Palumbo, Carlotta Pirrello, Silvia Farinati, Andrea Curioni, Aurélien Devillars, Margherita Lucchin, Gianni Barcaccia, Alessandro Vannozzi

**Affiliations:** 1 Department of Agronomy, Food, Natural Resources, Animals and Environment (DAFNAE), University of Padova, Agripolis, 35020 Legnaro, Italy; francesco.scariolo@unipd.it (F.S.); giovanni.gabelli@unipd.it (G.G.); gabriele.magon@unipd.it (G.M.); fabio.palumbo@unipd.it (F.P.); silvia.farinati@unipd.it (S.F.); andrea.curioni@unipd.it (A.C.); aurelien.devillars@phd.unipd.it (A.D.); margherita.lucchin@unipd.it (M.L.); gianni.barcaccia@unipd.it (G.B.); 2 Interdepartmental Centre for Research in Viticulture and Enology, University of Padua, Via XXVIII Aprile, 31015 Conegliano, Italy; 3 Research and Innovation Centre, Fondazione Edmund Mach, Via E. Mach 1, 38098 San Michele all’Adige, Italy; carlotta.pirrello@fmach.it

**Keywords:** PIWI, exocarp, RNA-seq, resistance, grapevine

## Abstract

PIWI, from the German word Pilzwiderstandsfähig, meaning “fungus-resistant”, refers to grapevine cultivars bred for resistance to fungal pathogens such as *Erysiphe necator* (the causal agent of powdery mildew) and *Plasmopara viticola* (the causal agent of downy mildew), two major diseases in viticulture. These varieties are typically developed through traditional breeding, often crossbreeding European Vitis vinifera with American or Asian species that carry natural disease resistance. This study investigates the transcriptional profiles of exocarp tissues in mature berries from four PIWI grapevine varieties compared to their elite parental counterparts using RNA-seq analysis. We performed RNA-seq on four PIWI varieties (two red and two white) and their noble parents to identify differential gene expression patterns. Comprehensive analyses, including Differential Gene Expression (DEGs), Gene Set Enrichment Analysis (GSEA), Weighted Gene Co-expression Network Analysis (WGCNA), and tau analysis, revealed distinct gene clusters and individual genes characterizing the transcriptional landscape of PIWI varieties. Differentially expressed genes indicated significant changes in pathways related to organic acid metabolism and membrane transport, potentially contributing to enhanced resilience. WGCNA and k-means clustering highlighted co-expression modules linked to PIWI genotypes and their unique tolerance profiles. Tau analysis identified genes uniquely expressed in specific genotypes, with several already known for their defense roles. These findings offer insights into the molecular mechanisms underlying grapevine resistance and suggest promising avenues for breeding strategies to enhance disease resistance and overall grape quality in viticulture.

## 1. Introduction

*Vitis vinifera* L. is considered one of the ancient crops in human history, with evidence of its consumption tracing back to the era when humans were still collectors [1]. This species belongs to the *Vitis* L. genus and includes the wild subspecies *V. vinifera* subsp. *sylvestris* and the cultivated subspecies *V. vinifera* subsp. *sativa* or *vinifera*. The belief that cultivated subspecies derive from the domestication of wild individuals is based on the genetic similarity between *sylvestris* and *vinifera*, as well as the presence of intermediate genotypes [2].

Until the first half of the 19th century, European viticulture relied on the cultivation of own-rooted grapevines. This changed dramatically when European vineyards encountered a crisis brought on by the introduction of native American pathogens and pests. European grapevines, which had never faced these threats, were particularly vulnerable. The root systems of European grapevines were highly susceptible to phylloxera infestation, a pest to which wild American grapevines were resistant [3]. In addition to pests such as phylloxera, other alien pathogens arrived from the New World, such as powdery mildew (PM) and downy mildew (DM), to impact viticulture. These challenges significantly affected grapevine production, necessitating new strategies and innovations in vineyard management for maintaining yield and health. The causal agents of DM and PM, respectively, the oomycete *Plasmopara viticola* and ascomycete *Erysiphe necator*, are obligate, biotrophic, and polycyclic pathogens that infect all green parts of their host plants [4,5]. *P. viticola* thrives in conditions of high relative humidity and mild temperatures [6,7], while *E. necator* optimally grows at 85% relative humidity and 26 °C [8]. Under favorable conditions, these pathogens undergo multiple cycles of clonal reproduction, causing severe damage to fruit quality and yield. To combat the impact of DM and PM, fungicides are frequently applied in vineyards. However, the use of these agrochemicals poses potential risks to human health and the environment [9]. Although *V. vinifera* is susceptible to these pathogens, American and Asian *Vitis* species show complete or partial resistance, impacting various stages of the pathogen’s life cycle. This involves reducing infection frequency, slowing tissue colonization, extending the latent period, and decreasing spore production, all without completely halting the infection [10]. The genetic basis of resistance traits in grapevine is typically identified using biparental mapping, where genetic maps are created by crossing two heterozygous parents and analyzing their segregating markers in the progeny (pseudo-testcross mapping strategy). An alternative method involves selfing populations. For quantitative trait loci (QTL) analyses, progenies are grouped based on inherited genotypes, and their phenotypes are compared to identify significant associations between traits and allelic variants [10].

To date, phenotyping and genotyping approaches to identify QTLs associated with resistance to pathogens such as *P. viticola* and *E. necator* have been applied to over 50 populations, with the development of several mapping populations produced by crossings between *V. vinifera* and different wild *Vitis* spp. [11,12,13,14], backcross individuals [15,16,17,18], and hybrid accessions [19,20,21,22,23]. Only one genome-wide association study (GWAS) incorporating both cross-generated individuals and natural grape cultivars has been conducted [24], and only two studies have utilized pedigree information to characterize resistance QTLs [25,26].

In general, progress in identifying resistance (R) loci has been steadily advancing. To date, 33 R loci against *P. viticola* (*Rpv*) and 15 against *E. necator* (*Ren* or *Run*) have been identified [10]. Of these, only a small number have been genomically characterized, including *Run1*, *Rdv1*, and *Rpv1* [23,24,27,28]. 

Since the advent of first-generation hybrids, extensive efforts have been dedicated to integrating resistance traits from American or Asian wild species into “elite” genetic material. This has led to hybrids combining disease resistance from wild grapevine species with desirable traits of traditional European *V. vinifera* varieties. Known as PIWI varieties (from the German “*pilzwiderstandsfähig*”, meaning fungus resistant), their number continues to grow through breeding programs. Currently, over 100 PIWIs are recognized, encompassing both established and newly developed cultivars. Ongoing research and breeding endeavors aim to enhance disease resistance, improve grape quality, and adapt to diverse climates.

Resistant varieties are now widely cultivated globally. Among the top resistant wine grape varieties, Concord is the most prevalent, covering 0.24% of the global vineyard area, primarily in Brazil, the USA, and Japan. Bianca follows with 0.22%, mainly in Russia, Hungary, and Moldova. Other notable varieties include Seyval Blanc, Regent, Chambourcin, Villard Noir, and Baco Noir, each accounting for 0.06–0.02% of the global vineyard area and cultivated across all continents [29,30].

Molecular biology and genomics have assisted in selecting resistant varieties by identifying *R* loci and establishing genotyping mapping populations. Moreover, the advent of omics technologies has further aided in understanding the molecular mechanisms behind resistance responses from a molecular perspective. On the other hand, high-throughput transcriptomic technologies have allowed a better and more comprehensive understanding of the transcriptional changes occurring during the grapevine response to DM or PM, thus pinpointing key defense regulators to be used as candidate markers of grapevine resistance. Resources aimed at this purpose could include genome-wide transcriptomic studies investigating the response of susceptible or resistant genotypes [31,32,33,34], the comparison between resistant and susceptible varieties [35,36,37,38,39,40,41], or the response of susceptible genotypes treated or not with resistance inducers [42,43].

Most of these studies were performed on leaf tissues pre- and post-pathogen inoculation. This is since, for example, concerning PM, the period of fruit susceptibility is in general small while green tissues like leaves are much more exposed to pathogen attack. After the onset of véraison, grape berries develop ontogenetic defense mechanisms that make them generally more resistant to PM and DM compared to green tissues. This process is part of the plant’s natural evolution to protect mature fruits, which are crucial for reproduction and seed dispersal.

Like other fleshy fruits, grapevine berries are complex organs composed of various tissues that develop according to both common and unique patterns. Grapes are divided into three distinct parts: exocarp (skin), mesocarp (flesh), and seeds [44]. Many of the relevant traits for table or wine grape quality seem to be specifically related to only one of the pericarp tissues. The grapevine skin consists of an outer epidermis (a single cell layer) and an inner hypodermis (a layer of one to seventeen cells). It is the specific site where terpenoid biosynthesis and carotenoid catabolism occur, and where cells lose their chlorophyll and transform into modified plastids during the véraison stage [45]. Additionally, it is the site of anthocyanin pigment synthesis during ripening, primarily responsible for berry and wine color [46,47]. Furthermore, the skin serves as an interface between the internal and external environments, shielding the latter from pathogen invasion and abiotic stresses. This requires a specialized composition of cell walls and layers of protective waxes [48,49]. In the literature, comparative transcriptome studies have been conducted on grape skin. These studies aim to better understand the genetic response related to specific traits influenced by both skin and flesh properties. For example, crispness and firmness are linked to mesocarp cell turgor pressure and skin resistance during various stages of development [50]. However, knowledge about the transcriptional landscape of the skin in resistant PIWI grape varieties is still limited compared to their elite parents.

The aim of this study was to investigate the transcriptional profile of the exocarp in mature berries from four PIWI varieties (two red and two white), and their noble parental varieties, to uncover molecular mechanisms that may contribute to the greater basal resistance observed in interspecific varieties compared to elite varieties. By employing tools such as WGCNA (Weighted Gene Co-expression Network Analysis) and tau analysis, we sought to identify genotype-specific gene clusters. Our findings highlight an increase in vesicular activity in the resistant varieties, which may play a role in their enhanced resistance.

## 2. Results and Discussion

### 2.1. Global Transcriptome Analysis of PIWI and Elite Varieties and Identification of Differentially Expressed Genes in Tolerant vs. Susceptible Red and White Varieties

RNA-seq on the NovaSeq 6000 platform produced a total of 760 million 2 × 150 bp reads, with an average of approximately 42 million reads per sample (Table S1). After filtering, 750 million reads were retained for further analysis and mapped to the 12X.v2 reference genome assembly using the VCost.v3 gene annotations [51]. On average, 88% of the reads mapped uniquely to the reference genome, equating to 36.5 million reads per sample. Summarized read count data are presented in Table S2. After removing annotations with fewer than 10 counts per sample, a principal component analysis (PCA) and a heatmap was generated to evaluate the concordance of each replicate within its group and to explore the similarities and differences between varieties.

**Figure 1:**
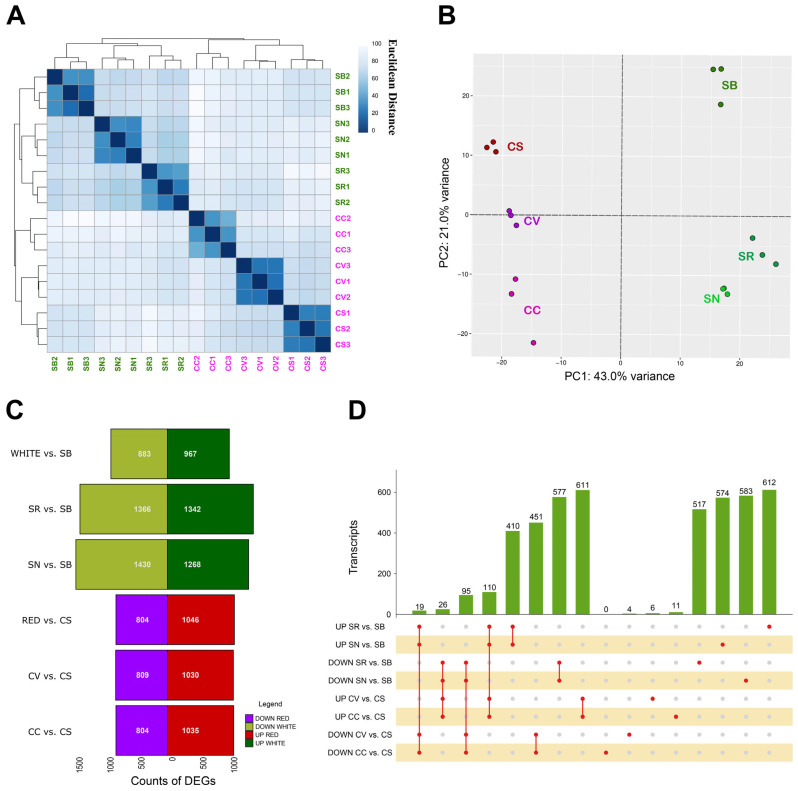
(**A**) Correlation matrix heatmap showing the Euclidean distance between samples based on normalized data obtained from 18 RNA-seq samples constituted of berry skin tissues of the CC, CV, CS, SR, SN, and SB varieties in the ripening (R) phase. A darker color indicates a stronger correlation. (**B**) PCA on normalized data obtained from 18 RNA-seq samples. Colors indicate different varieties considered. (**C**) The histogram shows the number of upregulated and downregulated DEGs in white and red PIWI varieties compared to their respective noble parents (SB for white and CS for red). It includes both cumulative comparisons of all PIWI varieties of the same color against their parental variety, as well as individual comparisons (e.g., SR vs. SB). (**D**) Upset plots visualizing the intersections amongst different groups of DEGs identified in pairwise comparisons. Single points indicate a private DEG identified in each group, whereas 2 to *n* dot plots indicate DEGs shared by 2 to n groups.

Replicates were organized in the heatmap by variety and clustered by color (Figure 1A), indicating greater similarity among the white varieties compared to the red ones. Similarly, in the PCA plot (Figure 1B), the first principal component (PC1) accounts for 43.0% of the variance, effectively separating the red and white varieties. The second principal component (PC2), explaining 21.0% of the variance, distinguished between the elite and PIWI varieties.

Using the DESeq2 dds function, differentially expressed genes (DEGs) were identified based on an adjusted *p*-value (padj) < 0.05 and a log2 fold change (log2FC) > |2|. DEGs from the white and red subsets were analyzed separately, as well as single comparisons between one PIWI and its elite parent (Supplementary Table S3). Analyses revealed distinct gene expression patterns between groups. The number of up- and downregulated DEGs was plotted for each dataset (Figure 1C). Notably, the highest number of DEGs (1430) was observed in the DOWN dataset SN vs. SB suggesting a significant downregulation of genes in the resistant variety compared to its parent. Conversely, the lowest number of DEGs (804) was found in the comparison of both red PIWI varieties (CC and CV) to the noble parental CS, indicating fewer changes in gene expression between these red varieties and their parent. In general, considering the single comparisons between the PIWI and relative elite parent, the white varieties exhibited a greater number of DEGs than the red varieties both in terms of up- and downregulation. This suggests that the white PIWI varieties might undergo more extensive transcriptional reprogramming compared to their red counterparts, possibly contributing to their differential basal resistance profiles. This aligns with previous findings that white PIWIs exhibit better adaptability to variable and stressful conditions, as well as earlier maturation [52].

Further investigation of the intersections between DEGs identified in single comparisons was performed using an upset plot (Figure 1D) showing both the shared and non-shared DEGs. In particular, the number of non-shared DEGs in the red varieties is very low compared to the numbers observed in the white varieties. This observation gains further significance when considering that on a genome-wide scale, the white varieties are more transcriptionally correlated with each other, as indicated in Figure 1A. Shifting to the analysis of DEGs shared among multiple samples, the most represented groups are those shared among the comparisons involving PIWI varieties that have the same noble parent. Specifically, these are the DEGs that are up- and downregulated in the comparisons between CV, CC, and CS, as well as those in the comparisons between SN, SR, and SB. This finding indicates that PIWI varieties derived from the same noble parent share similar transcriptional responses, reflected in the up- and downregulated DEGs. The shared DEGs among CV, CC, and CS suggest a conserved set of gene expression changes inherited from CS. Similarly, the DEGs shared among SN, SR, and SB highlight a common transcriptional profile influenced by Sauvignon blanc. This pattern suggests that the noble parent plays a significant role in shaping the transcriptional landscape of PIWI offspring, which could be leveraged in breeding programs to predict and enhance desirable traits [27,53].

Gene Set Enrichment Analyses of DEGs identified in pairwise comparisons indicate several ontological categories which appear to be enriched in the skin of resistant varieties in comparison to their noble parent (Table S4). Considering categories enriched with a significance level of *p* > 0.01, a notable observation is that, despite the higher number of DEGs, white PIWIs exhibited fewer enriched categories (58 in SN and 54 in SR) compared to the red ones (176 in CC and 177 in CV). Moreover, these varieties shared very few ontological categories, mainly related to “organic metabolic process” (GO:0005737) and “oxoacid metabolic process” (GO:0044281). In contrast, varieties with the same noble parent showed a much higher number of shared categories, especially the red berry varieties CC and CV, which shared almost all enriched categories (28.3%, 175 out of 178). The white berry varieties showed a lower number of shared categories (31.4%, 27 categories out of 86).

Among the significant categories, we focused on those with the highest fold change in enrichment to avoid categories that, although highly significant (low *p*-value), were too general to provide specific biological insights. Using this approach, we observed that both the CC and CV genotypes exhibit increased activity related to membrane and vesicle transport. This includes the “late endosome to vacuole transport via multivesicular body sorting pathway” (GO:0032511, FC~3), “endosome transport via multivesicular body sorting pathway” (GO:0032509, FC~3), “multivesicular body sorting pathway” (GO:0071985, FC~3), “AP-type membrane coat adaptor complex” (GO:0030119, FC~3), and “protein localization to chloroplast and vacuole” (GO:0072598 and GO:0072665). Conversely, the white varieties appeared to be enriched in categories related to the biosynthesis and metabolism of organic acids’ metabolism including the “organic hydroxy compound metabolic process” (GO:1901615), “monocarboxylic acid metabolic process” (GO:0032787), “oxoacid metabolic process organic acid metabolic process (GO:0043436), carboxylic acid biosynthetic process” (GO:0046394), and “carboxylic acid metabolic process” (GO:0019752). Also interesting is the enrichment of the “tripeptide transport” category. Looking at the enriched “unique” ontological categories in the DEGs of SR or SN, none stood out for their uniqueness, as many were associated with general processes related to primary or secondary metabolism (Table S4).

### 2.2. K-Means Associated Weighted Gene Co-Expression Network Analysis Indicates Gene Clusters Associated with PIWI Genotypes and Tolerance

**Figure 2:**
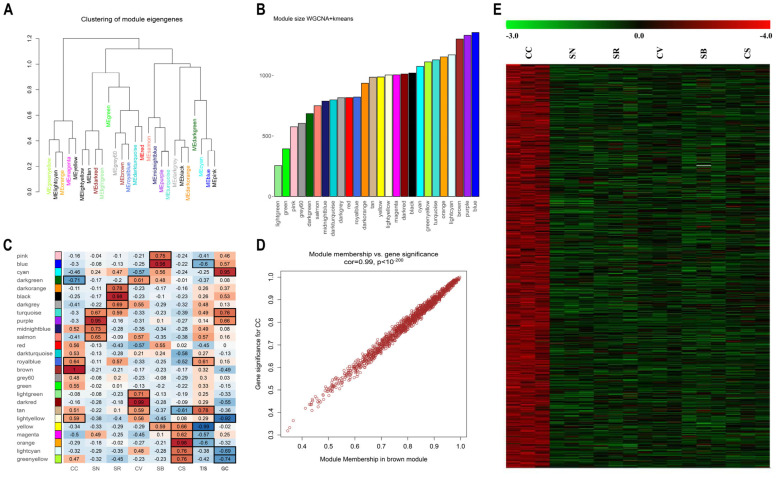
K-means-corrected WGCNA. (**A**) Cluster dendrogram of module eigengenes. Branches of the dendrogram group together eigengenes that are positively correlated. The merge threshold was set to 0.25: modules under this value were merged due to their similarity in expression profiles. (**B)** Bar graph showing the distribution of genes over the twenty-six modules identified. (**C**) Module-variety/trait association analysis. The heatmap shows the correlation between modules and varieties/traits. Each row corresponds to a module, whereas each column corresponds to a specific trait. The correlation coefficient between a given module and tissue type is indicated by the color of the cell at the row–column intersection and by the text inside the cells (squared boxes indicate significant *p*-values). Red and blue indicate positive and negative correlations, respectively. CC, Cabernet cortis; SN, Sauvignon nepis; SR, Sauvignon rytos; CV, Cabernet volos; SB, Sauvignon blanc; CS, Cabernet sauvignon; T/S, tolerance/susceptibility; GC, grape color. (**D**) Scatterplots of gene significance (GS) vs. module membership (MM) in the brown module associated with Cabernet cortis (CC). Genes highly significantly associated with a trait are often also the most important (central) elements of modules associated with the trait. (**E**) Heatmap visualizing gene expression within the brown module across all biological replicates of the six considered varieties, normalized using Z-scores.

Within plant biology, a plethora of seminal studies have harnessed WGCNA as a valuable tool, evident in diverse plant species including pineapple [54], strawberry [55], pear [56], and grapevine [57,58,59,60]. These studies highlighted the efficacy of WGCNA in unraveling the intricate regulatory networks and uncovering functionally related gene modules within plant systems. In our study, we sought to apply an enhanced version of WGCNA by integrating k-means clustering as an additional step. This powered approach, developed by Botía et al. [61], aimed to improve the precision of gene clustering within the co-expression network analysis compared to traditional WGCNA. Thus, our dataset comprising 23,847 genes was examined using an adapted version of the WGCNA R-package to detect gene co-expression modules. A soft-thresholding power β of 30 was applied to the matrix to establish a scale-free network. Modules were defined as groups of genes tightly connected to each other, exhibiting high correlation within the same module. Initially, a minimum module size of 30 was set, and modules with strongly correlated eigengenes (threshold: 0.25) were merged. A more stringent examination was conducted with a stricter cutoff (0.10). However, given the extensive number of modules obtained (103), these data are provided as supplementary data unless explicitly cited in the main text (Table S5). The eigengene, serving as the principal component, encapsulates the expression profiles of genes within a specific module. Twenty-six unique modules were delineated (threshold: 0.25), each denoted by a specific color and visualized in a hierarchical clustering dendrogram, illustrating their interrelations via individual module eigengenes (Figure 2A). The gene content of these modules varied from a minimum of 255 genes for the light green module to a maximum of 1355 for the blue one (Figure 2B). Subsequently, a correlation analysis was conducted between these 26 modules, the 6 varieties under investigation (CS, CC, CV, SB, SN, and SR), and 2 additional traits that distinguish the genotypes under study: the tolerance/susceptibility to pathogens (T/S) and the grape berry color (GC) (Figure 2C). The objective was to pinpoint gene modules distinctly associated with the diverse genotypes considered, to the tolerance/susceptibility, and to the color. For each variety/trait, at least one highly specific module was identified (correlation *p*-value < 0.01; Figure 2C, Table S6). However, in certain instances, multiple modules exhibited significant correlations, both positive and negative, with the same variety/trait or multiple varieties/traits displayed significant correlations with the same module. For example, CC was associated with the brown, dark green, light yellow, and royal blue modules, whereas the yellow module was simultaneously associated with SB, CC, and T/S. The most notable correlations between the module eigengene (ME) and variety were observed between the brown module and CC (r = 1, *p* = 6 × 10^−19^), the dark red module and CV (r = 0.99, *p* = 3 × 10^−14^), and finally, the black module and SR (r = 0.98, *p* = 6 × 10^−12^). The most notable correlations between T/S and GC traits were with the yellow (r = −0.99, *p* = 1 × 10^−15^) and tan (r = 0.95, *p* = 2 × 10^−9^) modules.

**Figure 3:**
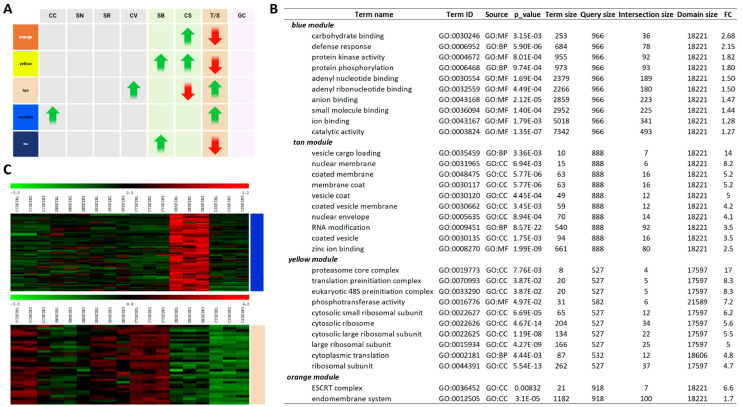
Modules contemporaneously associated with both tolerance/susceptibility and one or more grapevine varieties analyzed. (**A**) Table showing the orientation of correlations in all varieties/traits considered (CC, Cabernet cortis; SN, Sauvignon nepis; SR, Sauvignon rytos; CV, Cabernet volos; SB, Sauvignon blanc; CS, Cabernet sauvignon; T/S, tolerance/susceptibility; GC, grape color). Green arrows indicate a positive correlation between the specific module and the trait/genotype. Red arrows indicate a negative association between the specific module and the trait/genotype considered. (**B**) Gene Set Enrichment Analyses of the tan and blue modules showing the top 10 enriched categories based on fold change. The threshold *p*-value was set to 0.01 (**C**) Heatmap visualizing gene expression within the blue and tan modules across all biological replicates of the six considered varieties, normalized using Z-scores.

To delve deeper into the genetic composition of modules exhibiting notable correlations with the studied varieties/traits, two distinct network metrics, the gene significance (GS) and the module membership (MM), were employed. Module membership (MM) quantifies the degree of correlation between a gene’s expression profile and the respective module eigengene. Conversely, gene significance (GS) serves as an additional network parameter, often represented as the negative logarithm of a *p*-value, offering an estimation of a gene’s biological relevance. The greater the absolute value of GSi, the greater the biological significance of the *i*-th gene. In essence, genes demonstrating higher GS and MM values hold increased significance with respect to the phenotypical trait [58]. Consequently, a specific module exhibiting significantly interconnected MM or GS values associated with the tolerance (susceptibility) trait may play a pivotal biological role in resistance mechanisms. Among the considered modules, the brown (CC; cor = 0.99, *p* < 1 × 10^−200^), purple (SN; cor = 0.89, *p* < 1 × 10^−200^), black (SR; cor = 0.97, *p* < 1 × 10^−200^), dark red (CV; cor = 0.97, *p* < 1 × 10^−200^), blue (SB; cor = 0.97, *p* < 1 × 10^−200^), orange (CS; cor = 0.96, *p* < 1 × 10^−200^), yellow (tolerance; cor = −0.98, *p* < 1 × 10^−200^), and cyan (GC; cor = 0.86, *p* < 1 × 10^−200^) modules displayed the strongest correlations between MM and GS (Figure S1). A Gene Set Enrichment Analysis (GSEA) was performed to ascertain the occurrence of enriched categories related to biological process (BP), molecular function (MF), or cell component (CC) ontologies in all modules obtained by the analysis (Tables S7 and S8). One particularly intriguing aspect pertains to those modules displaying significant correlations with both the T/S trait and one or more examined genotypes, totaling 5 under a 0.25 cutoff and increasing to 18 using a 0.10 cutoff (Table S6). These modules consistently demonstrate a distinct behavior: when associated to the T/S and a susceptible genotype (CS or SB), the correlations exhibit inverse tendencies, whereas their associations with the tolerance trait and a resistant variety consistently display aligned correlations (Figure 3A).

No significant ontologies indicating enrichment were identified within the royal blue module, which exhibited associations with both CC and T/S. Conversely, when examining the tan module, positively correlated with both CV and T/S, a substantial number of enriched categories were evident (Figure 3B). As a general observation, most enriched genes within the top 10 enriched categories ordered by fold change (FC) collectively represent the machinery involved in various stages of vesicle trafficking, including cargo selection, vesicle formation, and membrane fusion. This observation is consistent with the enrichment analyses performed on the DEGs in the pairwise correlations. The fact that two independent analyses highlighted the same category of genes supports the notion that vesicle transport might be a process that specifically distinguishes the red PIWI varieties, particularly CC, and can be associated, at least based on the WGCNA, with tolerance.

**Figure 4:**
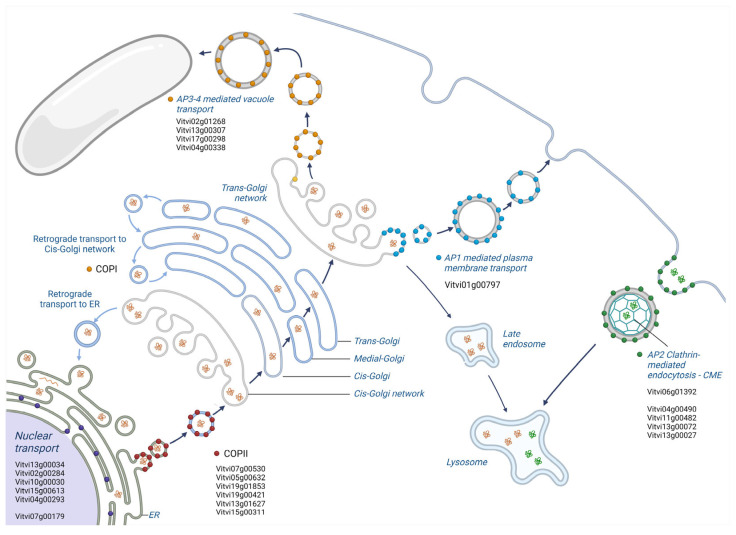
Vesicle transport pathways in plants. COP-II vesicles mediate cargo transport from the ER to the cis-Golgi, while COP-I traffics the cargo from the Golgi to the ER and intra-Golgi as well. Clathrin-mediated endocytosis (CME) is the primary mechanism by which eukaryotic cells internalize extracellular or membrane-bound cargoes and it plays crucial roles in plant–microbe interactions Clathrin-coated vesicles (CCVs) are involved in the flow of cargo from the plasma membrane and trans-Golgi network to endosomes and retromers. Grapevine genes found to be enriched in the tan module are indicated in proximity to the related transport pathway.

Of particular interest was the enrichment observed in a gene class associated with specific adaptor complexes’ proteins (APs) and with the formation of clathrin-coated vesicles. Adaptor protein (AP) complexes are evolutionarily conserved vesicle transport regulators that recruit coat proteins, membrane cargoes and coated vesicle accessory proteins and finely tune their sorting within the cell [62]. In mammalian cells, the AP-1, AP-2, and AP-3 complexes operate in concert with the scaffolding molecule clathrin to generate clathrin-coated vesicles (CCVs), which are involved in various cellular functions such as receptor-mediated endocytosis, protein sorting, and the transport of molecules within the cell. Clathrin-mediated endocytosis (CME) is the primary mechanism by which eukaryotic cells internalize extracellular or membrane-bound cargoes and play crucial roles in plant–microbe interactions [63,64,65]. CME is essential for immune responses mediated by pattern recognition receptor (PRR) kinases, facilitating the internalization and degradation of activated extracellular PRRs in the vacuole [66]. Examples include PRRs such as PEP RECEPTOR 1/2 (PEPR1/2) recognizing endogenous plant peptides, EF-TU receptor (EFR) recognizing bacterial Elongation Factor TU, FLAGELLIN-SENSING 2 (FLS2) recognizing flagellin (flg-22) as a pathogen-associated molecular pattern (PAMP), and the Cf-4 receptor-like protein recognizing the Cladosporium fulvum *Avr4* avirulence effector [66,67,68]. Another group of highly represented genes including *Vitvi07g00530*, *Vitvi05g00632*, *Vitvi19g01853*, *Vitvi19g00421*, *Vitvi13g01627*, and *Vitvi15g00311* encode for subunits of the cytosolic proteins Sec23, Sec24, and Sec31, which are constituents of the COPII complex involved in intracellular protein transport from the endoplasmic reticulum (ER) to the Golgi apparatus [69]. Additionally, *Vitvi07g00179*, a *RANGAP1* ortholog, regulates RAN GTPase activity, which indirectly influences vesicle trafficking (Figure 4).

Although a significant correlation was only found between the tan module and the CV genotype, resistant varieties show a higher expression of vesicle cargo and clathrin-related genes compared to susceptible ones in all the PIWIs analyzed, except for SN (Figure 3C). The resistant varieties exhibit an enrichment of genes involved in vesicular trafficking activity not only between the plasma membrane and internal organelles such as the nucleus, Golgi, and endoplasmic reticulum but also among these organelles. This heightened translocation activity may be attributable to various biological processes already associated with resistance, such as (i) the transport of defense compounds including defense-related molecules such as phytoalexins, antimicrobial peptides, and secondary metabolites; (ii) the regulation of antioxidant systems, important for detoxifying reactive oxygen species (ROS) and protecting cells from oxidative damage; (iii) the maintenance of a higher level of antioxidant capacity in the berry skin, which can contribute to its resilience against biotic and abiotic stresses; (iv) the modulation and strengthening of cell wall integrity; (v) the regulation of hormone signaling networks in plants, including those mediated by salicylic acid (SA), jasmonic acid (JA), and ethylene (ET), which play key roles in plant defense responses. The resistant variety may exhibit constitutive expression of vesicle cargo and clathrin-related genes to modulate hormone signaling pathways in the berry skin, priming it for rapid and effective defense activation upon pathogen attack [70,71,72,73,74,75,76].

Another interesting observation, which could seem counterintuitive at first glance, is the enrichment of defense-related genes in the blue module, which is associated to the susceptible variety SB and is anticorrelated with the T/S trait (Figure 3B,C). Over the 78 defense-related genes in the blue module are RPS genes, R protein PRF disease resistance genes, pathogen-related proteins, MLA10, etc. There could be several reasons why we detected an enrichment of defense genes in the susceptible grapevine variety compared to the resistant one: (i) the susceptible variety might have evolved to maintain a higher baseline expression of defense genes as a proactive strategy against potential threats. This could be due to historical interactions with pathogens or environmental stresses in its natural habitat; (ii) the susceptible variety might have deficiencies in other aspects of its defense system, leading to a compensatory upregulation of certain defense genes to mitigate its vulnerability. In contrast, the resistant variety may have more robust defense mechanisms overall, requiring less reliance on constitutive expression of defense genes; (iii) the constitutive expression of defense genes could also be influenced by environmental factors such as soil composition, climate, or microbial communities present in the vineyard. The susceptible variety might perceive these factors as potential threats and maintain higher levels of defense gene expression as a preemptive response; (iv) there could be trade-offs between growth/development and defense in the susceptible variety, where resources are allocated to defense at the expense of other physiological processes. This could result in the constitutive expression of defense genes even in the absence of infection.

### 2.3. PIWI Varieties Show Absolutely and Highly Specific Genes Which Are Involved in Disease Resistance

**Figure 5:**
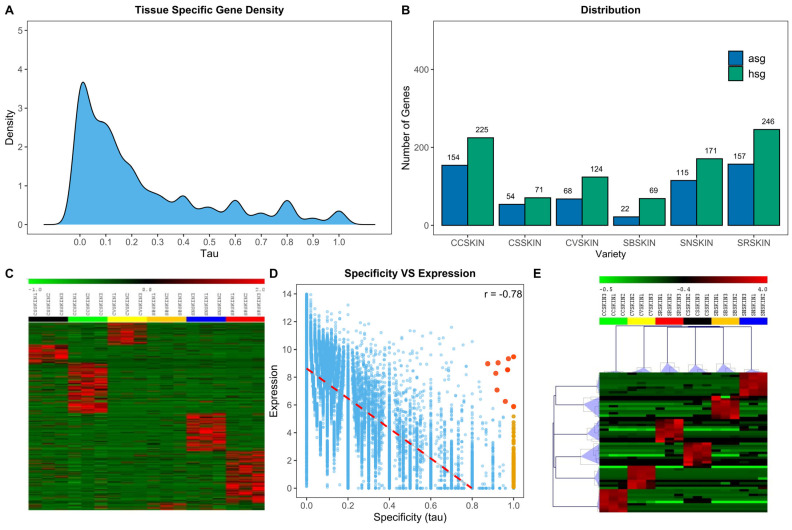
Identification of absolutely specific genes in different grapevine varieties. (**A**) Distribution of the variety-specificity tau parameter over the 23,847 genes considered. (**B**) Bar graph showing the distribution of absolutely specific genes (ASG; tau = 1) and highly specific genes (HSG; tau > 0.85) over the six varieties considered. (**C**) Heatmap illustrating the expression of ASG in all biological replicates of the six varieties considered (Z-score normalized). (**D**) Scatterplot illustrating the relation/negative correlation r = −0.78) between specificity (tau) and expression in Sauvignon nepis. Blue dots represent all genes considered in the analysis, orange dots represent ASG in S. nepis, and red dots indicate the top optimal genes for S. nepis based on the score value. (**E**) Heatmap showing the expression of the top 10 optimal genes identified over the six varieties considered.

In addition to the WGCNA analysis, we employed an algorithm commonly utilized in transcriptomic investigations involving animals or humans. However, it had previously been applied in the analysis of tissue-specific genes within the *P. noir* flower, as described by [58]. This algorithm, denoted as the tau (τ) algorithm, was employed to assess the tissue-specificity level of each predicted gene within a given genome [77]. Following the quantile normalization of 23,847 genes, which were selected based on their expression levels, and the subsequent creation of BIN profiles, the τ algorithm was utilized to assign a value ranging from zero (indicating constitutive expression across all or most tissues) to one (indicating absolute specificity for a particular tissue) to each gene. The distribution of τ values across the entire gene set is depicted in Figure 5A.

In summary, 906 genes exhibited a high degree of specificity (referred to as highly specific genes, HSGs, with τ > 0.85), and among them, 570 were identified as absolutely specific genes (absolutely specific genes, ASGs, with τ = 1). It is important to note that the τ value only characterizes the “specificity” of a gene. To ascertain the specific tissue to which a gene is exclusive, we computed the τ expression fractions (τef). Among the various grapevine varieties studied, SR displayed the highest count of HSG (246) and ASG (157). Conversely, SB exhibited the lowest number of HSG and ASG values (69 and 22, respectively), as illustrated in Figure 5B. Figure 5C reports a heatmap showing the Z-score normalized expression of ASGs in all biological replicates for each variety. As a general observation, the elite grapevine varieties Cabernet sauvignon and Sauvignon blanc displayed relatively lower counts of ASG and HSG genes. Detailed lists of HSG and ASG for each grapevine variety can be found in Table S9.

As illustrated in the scatter plot depicted in Figure 5D, the expression levels of genes tend to decrease as specificity (tau) increases. This implies that many highly specific genes associated with a particular tissue might exhibit low expression levels within that tissue and nearly absent expression in others. Prioritizing genes based on both their expression and specificity proves advantageous, pinpointing a cohort of genes that display significant specificity to the tissue, boast ample expression levels (beneficial for lab experimentation), and demonstrate minimal expression in other tissues, thereby mitigating off-target effects. To accomplish this, we compiled quantile-normalized expression data and specificity metrics for all genes identified through tau analysis. Utilizing this dataset, we devised a scoring mechanism wherein each gene was assigned a score ranging from zero to two. This score was computed by summing its tau expression fraction value (τ_ef_) and its expression value normalized within the zero to one range. Subsequently, for each tissue, we identified the top 10 genes based on their score values, representing genes that exhibit both heightened expression and specificity (Figure 5E). While the complete list of these genes is available in Table S10, Table 1 explicitly showcases the foremost gene ranked highest for optimal specificity within each distinct tissue. The tau expression fractions of the 10 optimum genes are visually depicted in Supplementary Figure S2. The absolute specificity of certain genes for a particular genotype may be attributed to a mapping issue due to variations in the coding sequence affecting read mapping and consequently the RNA-seq results. Unfortunately, genomic sequences of the resistant varieties are not available; however, sequences of Cabernet sauvignon and Sauvignon blanc are accessible (www.grapegenomics.com). To dispel any doubts regarding mapping, the coding sequences (CDSs) of the top 10 significantly expressed genes of each resistant variety were conserved and their CDS in the elite varieties were aligned with those of the PN40024 reference genome. Although such a comparison does not provide information about the lack of expression of a particular gene in other resistant varieties, it can offer insights into the absence of expression in elite ones. For instance, the GDSL lipase gene (*Vitvi07g02026*), exclusively expressed in Cabernet volos, shows no polymorphism in the noble parent C. sauvignon or S. blanc. The lack of expression of this gene in these varieties is thus not attributable to polymorphism in the coding region but to a different level of basal expression dictated by distinct regulatory mechanisms.

Several genes among the top 10 ASGs in resistant varieties warrant particular attention. One such gene is *Vitvi07g02026*, which is specifically expressed in CC and encodes a GDSL lipase (VvGELP21). GDSL esterases/lipases are a subclass of lipolytic enzymes that play crucial roles in plant growth and development, stress response, and pathogen defense. Numerous studies have demonstrated the involvement of this gene class in defense mechanisms against biotic stresses. For instance, Ji et al. (2023) demonstrated the role of a GDSL esterase/lipase, GELP1, in defending apple leaves against *Colletotrichum gloeosporioides* infection [78]. Additionally, the overexpression of *AtGDSL1* enhances resistance to *Sclerotinia sclerotiorum* in oilseed rape by modulating salicylic acid (SA)-dependent and jasmonic acid (JA)-dependent pathways. This overexpression leads to increased phosphatidic acid accumulation and the activation of downstream stress response pathways following Sclerotinia infection [79]. In Cabernet volos, *Vitvi11g01637* encodes for a MLA R protein (MLA10), a class of protein involved in PM resistance in barley [80].

In Sauvignon nepis, several ASGs can be associated with pathogen resistance. *Vitvi09g01181* encodes for a HcrVf1 protein which is known for its role in providing resistance against apple scab, a fungal disease [81]. The specific pression of an *HcrVf2-like* gene in SN berries implies that this gene may play a crucial role in the plant’s defense mechanisms, being involved in recognizing and responding to pathogen attacks and thereby enhancing the plant’s ability to resist diseases.

*Vitvi18g02399* is an Avr9 elicitor response factor. Genes belonging to the same family were found to be upregulated in PM infection in infected fruits of the susceptible *V. vinifera* cv. Carignan [34] and were reported to regulate the hypersensitive response in grapevine leaves [82,83]. Another interesting gene found to be exclusively expressed in the berry skin of SN is *Vitvi13g02352*, encoding for the disease-resistance protein RGA2 (Resistance Gene Analog 2), which was found to play roles in conferring resistance to downy mildew [84]. As an NBS-LRR protein, RGA2 detects specific pathogen-associated molecular patterns or effector proteins, triggering a defense response. Upon recognition, RGA2 activates signaling cascades that produce reactive oxygen species (ROS) and calcium ions, leading to localized cell death (hypersensitive response) to limit pathogen spread. Additionally, it induces the production of antimicrobial compounds and strengthens cell walls, enhancing overall plant immunity.

In conclusion, beyond specific metabolic pathways such as those related to vesicular transport identified in resistant red varieties, we have identified a series of genes (for a complete list, see Table S10) associated with resistance responses and expressed exclusively in resistant varieties. These genes could become targets for genetic improvement techniques, such as cisgenics or genome editing, of elite susceptible varieties, pending functional characterization.

## 3. Materials and Methods

### 3.1. Plant Material and Sample Collection

To examine the transcriptional landscape of the exocarp in ripe berries from PIWI varieties and their noble parents, we conducted an RNA-seq analysis on 18 berry samples collected from field-grown plants, located in a graveyard in Fossalon di Grado (Udine, Italy). The plants studied included two red-berried PIWI varieties (Cabernet cortis, CC, and Cabernet volos, CV), two white-berried PIWI varieties (Sauvignon rytos, SR, and Sauvignon nepis, SN), and their respective noble parents: Cabernet sauvignon (CS) and Sauvignon blanc (SB). The berries used for the transcriptomic analysis were collected at full technological maturity, corresponding to E-L Stage 38 of the Eichhorn–Lorenz scale. Maturity was determined based on sugar content (°Brix) and the visual assessment of full ripeness, ensuring that the fruit had reached complete development before sampling. Moreover, no disease symptoms were observed on plants or berries during sampling. We collected samples in three biological replicates, with each replicate comprising 30 berries from three different field-grown plants per variety. The PIWI varieties considered in this study possess QTLs mediating partial resistance to grapevine DM and PM. The cultivar Cabernet cortis (CC) harbors the combination of *Rpv3.3* and *Rpv10* loci which concerns the resistance to DM, and *Ren3* and *Ren9* loci for the resistance to PM. It was obtained in 1984 by Norbert Becker by crossing Cabernet sauvignon and the Solaris variety. The DM and PM resistance loci were transmitted from a Solaris cv initially introgressed from *V. amurensis*, a wild species of the Asian Vitis gene pool. The cultivar Cabernet volos (CV) was obtained by the University of Udine and Institute of Applied Genetics (IGA) in 2002, crossing Cabernet sauvignon (CS) and Kozma 23-2—a *Vitis* interspecific crossing between Bianca and Sremski karlovci 77 4-5. The cultivars Sauvignon nepis (SN) and Sauvignon rytos (SR) were obtained by the University of Udine and Institute of Applied Genetics (IGA) in 2002, crossing Sauvignon blanc (SB) and Bianca—an interspecific hybrid whose lineage is made up of *V. vinifera*, *V. rupestris*, *V. lincecumii*, *V. labrusca*, and *V. berlandieri*.

### 3.2. RNA Purification, Library Preparation, and Sequencing

Berries were sectioned, and their skins were dissected using scalpels while being kept frozen with liquid nitrogen. The isolated exocarps were processed for RNA purification using the Spectrum™ Plant Total RNA Kit (Sigma-Aldrich, St. Louis, MO, USA) following the manufacturer’s instructions. For RNA extracts of low quality due to contaminants, further purification was performed using lithium chloride (LiCl) precipitation. This involved the addition of a 2.5 M LiCl solution to the extracted RNA, followed by overnight incubation at 4 °C. The next day, samples were centrifuged at 14,000 RPM for 15 min at 4 °C. The supernatant was carefully removed, and the samples were washed with 500 µL of 70% ethanol prepared in DEPC (diethyl pyrocarbonate) water. Nanodrop (Thermo Fisher Scientific, Wilmington, DE, USA) (quantity and purity) and Labchip GX (PerkinElmer, Waltham, MA, USA) (quality) were used to assess the quality and quantity of the isolated RNA. Then, cDNA libraries were constructed using ≥600 ng RNA per sample with the Hieff NGS Ultima Dual-mode mRNA Library Prep Kit for Illumina (Yeasen Biotechnology (Shanghai) Co., Ltd., Shanghai, China), following the manufacturer’s recommendations. The effective concentration of the library was determined to be at least 2 nM. The resultant sequencing libraries were then submitted for sequencing on the Illumina NovaSeq 6000 (Illumina, San Diego, CA, USA) platform, in PE150 mode. All raw reads were deposited in the NCBI SRA database with accession numbers SRRXXXX–SRRXXXXX.

### 3.3. RNA-seq Analysis

Read trimming and quality filtering were performed using the fastp v.0.20.1 software with default parameters [85]. Filtered reads were mapped on the 12x.v2 assembly of the PN40024 reference genome using the VCost.v3 annotation [51]. The reads alignment and counting were conducted using STAR software version 2.7.11a [86] using the following parameters: *outMultimapperOrder* = Random, *outSAMmultNmax* = 10, *outWigStrand* = Stranded, *outWigNorm* = None, *quantMode* = GeneCounts, *twopassMode* = Basic. The resulting counts were normalized with R/DESeq2 v.1.34.0 [87] with the method of the median of ratios.

### 3.4. Differential Gene Expression Analysis

Statistical analysis of the mapped reads was performed using the reads count as computed by the aligner STAR, normalized through the median of ratios method, and the R package DESeq2 [87]. Once the normalized reads counts and the DESeq dataset were created, a first filtering was performed to remove transcripts with less than 10 mapped reads before DEGs identification. Differentially expressed genes were identified considering two variables: (i) the grapevine variety typology (“noble” or “PIWI”), and (ii) the berry color (“White” and “Red”). The DEGs analysis was performed using the *dds* function from the DESeq2 package, and those with an adjusted *p*-value < 0.05. This first screening was performed for the overall dataset, and for the sub-datasets, focused on red and white varieties, which compared PIWI versus elite varieties separately (e.g., Cabernet volos vs. Cabernet sauvignon; Sauvignon nepis vs. Sauvignon blanc). Principal component analyses (PCAs) and heatmaps were computed through *vst* and *pheatmap* functions, respectively, to verify the concordance between replicates of each variety and to investigate the relationships between them. Upset plots to represent the private and shared DEGs between different groups were created using the upsetR package in RStudio [88].

The Gene Ontology enrichment analysis was performed using the R package gprofiler2 (v.0.2.3) using the default parameters and no custom genes background, since the gene excluded from the analyses had no sensible expression. The functional annotation of *Vitis vinifera* is present in the g:Profiler database under the name “vvinifera” [89].

### 3.5. K-Means Corrected Weighted Gene Co-Expression Network Analysis

The normalized expression indexes of all the samples were used to build a co-expression network and a first hierarchical clustering of the genes using the R package WGCNA v.1.72.5 [90]. The following parameters were passed to the function net: TOMType = “signed”, minModuleSize = 25, power = 30. The optimal power value was defined with the function pickSoftThreshold of the same package. To obtain two different levels of sensibility (i.e., smaller and more uniform modules or bigger and more generally descriptive ones), two different values of the parameter mergeCutHeight were adopted, 0.1 and 0.25, respectively. All the other parameters were kept to the default setting. The genes modules were then used as inputs for a k-means clustering algorithm from the package CoExpNets, wrapped by the function applyKMeans [61]. The parameters adopted were the defaults except for n.iteration = 50 and excludeGrey = TRUE. A linear correlation between the eigengene of each module and a series of logical vectors describing the studied character of the samples (i.e., the cultivar, the tolerance/resistance, and the color of the berries) was used to identify the modules associated and therefore of interest. The dedicated functions in the WGCNA package were used to further investigate these modules in terms of gene significance (GS, the linear correlation between the expression vector of the genes and the logical vector of the studied characters) and module membership (MM, a measure of centrality of the gene in the module co-expression network). A Gene Ontology enrichment analysis was performed on the genes composing the modules with the same procedure described in the previous paragraph.

### 3.6. Identification of Highly Specific Genes (HSG) and Absolutely Specific Genes (ASG) by Means of Tau Analysis

The index τ (tau) was used as an indicator of the tissue-specificity level of the expression of a gene [77]. Its range is from zero (for widely expressed genes) to one (for tissue/stage-specific genes). To calculate it, the tispec R package v0.99.0 was used. The reads counts were normalized using the TPM method with the R package bioinfokit v2.0.3. All the genes with less than 1 TPM in any tissue were then filtered out. To allow comparisons between tissues, the values were logarithm transformed, setting all the negative values to 0, and a quantile normalization, assigning to every gene a BIN value from 0 (lowest expression) to 10 (highest expression). The τ index of every gene was computed as follows:

$$\tau =\frac{\sum_{i=1}^{N}\left(1-x_{i}\right)}{N-1}$$

where N is the number of tissues and x_i_ is the expression value normalized by the highest expression. Genes expressed in a single tissue, with a τ of 1, are here referred as absolutely specific genes (ASGs), while we define as highly specific genes (HSGs) genes with a τ higher or equal to 0.85. A second index, a function of the normalized expression of the gene, was called τ_ef_ and calculated as follows:

$$\tau_{ef}=\tau \frac{qn}{max}$$

where qn is the quantile normalized expression and max is the highest quantile normalized expression. For every gene in every tissue, the sum of τ and τ_ef_ was called the τ-score and used as a proxy of the relevance of the gene in that tissue.

## 4. Conclusions

In this study, we used a transcriptomic approach to identify key metabolic pathways and specific genes that differentiate the basal defense mechanisms of resistant and susceptible grapevine varieties. Instead of focusing on infected versus uninfected plants, we analyzed the molecular baseline to uncover critical differences that prime certain varieties for enhanced tolerance. The berry skin, as the first barrier against pathogens, was chosen for this study due to its central role in defense processes, including pathogen recognition, signaling, and the activation of protective responses.

Our analyses revealed gene clusters associated with resistance and susceptibility, with a significant upregulation of vesicular transport mechanisms in red PIWI varieties. These processes, involving vesicle formation and cargo transport, have been linked to pathogen defense across various plant species. Their role in enhancing resistance likely lies in the more efficient trafficking of antimicrobial compounds or signaling molecules, providing a mechanistic insight into the improved resistance profile of these varieties.

We also identified a set of highly specific genes (HSGs) and absolutely specific genes (ASGs) uniquely expressed in PIWI varieties, likely playing key roles in basal defense. The exclusive presence of these genes in resistant varieties underscores their importance in adaptation to environmental stress and pathogen pressure. As PIWI varieties stem from breeding programs incorporating wild genomic contributions, these wild alleles likely harbor regulatory sequences or genes that modulate basal defense. This could explain the superior resistance observed in PIWI varieties.

While our study provides valuable insights into resistance mechanisms, further validation is needed. Some of the identified genes, especially those linked to vesicular transport and defense responses, present strong candidates for functional characterization through knock-out or knock-down experiments. Such studies would confirm their precise role in resistance and could lay the groundwork for future breeding strategies.

Additionally, our findings emphasize the complex nature of quantitative trait loci (QTLs) associated with resistance traits. Identifying regulatory networks and variety-specific gene expressions is crucial for understanding the genetic basis of resistance. The genes identified in this study could serve as promising candidates for Marker-Assisted Selection (MAS) in breeding programs, allowing for the introgression of resistance-associated traits into elite grapevine cultivars.

Looking forward, these results open avenues for future molecular studies and genetic manipulation aimed at producing highly resistant grapevine varieties. Beyond traditional breeding, these genes could be targeted by advanced biotechnological approaches such as assisted evolution or cisgenesis. These methods would enable the transfer of beneficial traits from PIWI varieties into elite cultivars without introducing foreign DNA. By adopting these techniques, the viticulture industry could reduce its dependence on chemical treatments and enhance crop resilience in the face of evolving pathogen threats.

## Figures and Tables

**Table 1:** List of the ten best ranking genes based on a score value (0–2) corresponding to the sum of the quantile normalized expression of a given gene and its tau expression factor.

Gene ID V3	Gene ID V2	Tau	Score	Mean Exp	Functional Annotation
*Cabernet sauvignon*					
Vitvi01g02070	VIT_01s0127g00910	0.95	1.265	56.55	AERO1
Vitvi10g00903	-	1.00	1.249	23.71	Leucin-rich repeat protein kinase
Vitvi01g02281	VIT_01s0010g04010	1.00	1.182	9.77	Unknown protein
Vitvi10g02153	-	1.00	1.133	5.16	-
Vitvi07g00496	VIT_07s0005g02310	0.93	1.133	12.47	EXPA17
Vitvi05g02072	-	0.93	1.130	11.93	-
Vitvi10g00183	-	1.00	1.125	4.63	-
Vitvi10g02415	VIT_00s2472g00010	0.85	1.112	27.96	Enhancer of mR-decapping protein 4
Vitvi01g02068	-	1.00	1.111	3.84	-
Vitvi10g02416	-	1.00	1.098	3.25	-
*Cabernet cortis*					
Vitvi07g02026	VIT_07s0130g00200	1.00	1.423	238.36	*VvGELP21*-Lipase GDSL
Vitvi19g00082	VIT_19s0014g01060	1.00	1.417	220.86	Sesquiterpene synthase
Vitvi09g01530	VIT_09s0002g01980	0.96	1.325	113.34	Myosin-like protein XIK
Vitvi11g01266	VIT_11s0052g01230	0.93	1.322	153.64	Xyloglucan endotransglucosylase/hydrolase 23
Vitvi09g01648	-	0.96	1.309	91.87	-
Vitvi08g02288	VIT_08s0007g04580	0.86	1.303	319.66	UGT73C2 (UDP-glucosyl transferase 73C2)
Vitvi19g00324	VIT_19s0014g04000	1.00	1.285	40.05	Curculin (mannose-binding) lectin
Vitvi12g02451	VIT_12s0134g00650	1.00	1.274	34.63	Anthocyanin 5-aromatic acyltransferase
Vitvi19g01982	VIT_19s0014g05140	0.95	1.271	64.65	-
Vitvi15g00285	VIT_15s0045g00270	1.00	1.265	30.67	Serine/threonine-protein phosphatase BSL3
*Cabernet volos*					
Vitvi14g00668	VIT_14s0036g00990	0.86	1.432	1814.13	Polyubiquitin (UBQ4)
Vitvi11g01637	VIT_11s0052g00270	0.85	1.346	652.97	R protein MLA10
Vitvi11g00879	VIT_11s0065g00040	0.89	1.331	321.15	CYP706A12
Vitvi04g00345	VIT_04s0008g04000	0.87	1.295	256.39	Unknown
Vitvi03g01478	VIT_03s0038g04230	0.88	1.240	103.63	Dihydroflavonol 4-reductase
Vitvi08g02374	VIT_08s0007g07760	0.93	1.175	21.69	Polygalacturonase PG1
Vitvi16g01677	-	1.00	1.169	8.42	-
Vitvi08g00789	VIT_08s0058g00650	1.00	1.161	7.72	Aldose reductase
Vitvi11g01568	VIT_11s0065g00740	1.00	1.142	6.04	A -phase-promoting complex subunit 8
Vitvi01g01642	VIT_01s0010g03550	1.00	1.140	5.97	Nuclear transcription factor Y sub-B related
*Sauvignon blanc*					
Vitvi04g00029	VIT_04s0008g00370	0.85	1.156	57.39	Clavata1 receptor kinase (CLV1)
Vitvi06g01648	VIT_06s0004g02550	0.85	1.150	52.71	Kiwellin Ripening-related protein grip22
Vitvi04g00021	VIT_04s0008g00300	0.85	1.147	50.92	Clavata1 receptor kinase (CLV1)
Vitvi09g01948	-	0.85	1.140	45.92	HcrVf2 protein
Vitvi03g00460	VIT_03s0063g01000	0.85	1.127	39.01	Blue (type 1) copper domain
Vitvi07g01769	VIT_07s0031g00850	0.87	1.094	20.80	Patatin
Vitvi01g01852	VIT_01s0011g00990	0.87	1.080	17.09	RPM1
Vitvi10g00005	VIT_10s0116g00150	0.87	1.078	16.85	Receptor kinase RK20-1
Vitvi00g02077	VIT_00s0895g00010	0.87	1.061	13.52	Glucan 1,3-beta-glucosidase
Vitvi16g02124	VIT_00s0294g00100	1.00	1.060	2.29	BR insensitive 1 receptor kinase 1
*Sauvignon nepis*					
Vitvi09g01181	VIT_09s0018g00780	1.00	1.506	710.79	HcrVf1 protein
Vitvi18g02399	VIT_18s0089g01040	0.97	1.474	647.26	Avr9 elicitor response
Vitvi10g01863	VIT_10s0003g03530	0.97	1.427	382.88	Lupeol synthase
Vitvi10g01875	VIT_10s0003g03650	0.97	1.427	381.69	Beta-amyrin synthase
Vitvi13g02352	VIT_13s0139g00190	0.92	1.408	529.73	Disease resistance protein RGA2
Vitvi12g02393	VIT_12s0059g01790	0.91	1.356	321.51	Caffeic acid O-methyltransferase
Vitvi02g00721	VIT_02s0012g01610	0.87	1.355	506.49	Beta-1,3-gluca -se precursor
Vitvi03g01757	-	1.00	1.314	63.03	-
Vitvi03g00910	VIT_03s0167g00050	0.92	1.298	140.99	Conca-valin A lectin
Vitvi16g00665	VIT_16s0022g00420	0.96	1.294	81.21	SRG1 oxidoreductase
*Sauvignon rytos*					
Vitvi01g01410	-	1.00	1.326	67.07	-
Vitvi08g00957	VIT_08s0040g00920	0.87	1.280	207.61	Glutathione S-transferase 25 GSTU7
Vitvi14g00080	VIT_14s0060g00990	1.00	1.269	31.94	Unknown
Vitvi13g02566	VIT_13s0156g00390	1.00	1.267	31.10	Myb family
Vitvi18g03265	VIT_18s0089g01000	0.95	1.256	51.80	F-box family protein
Vitvi15g01230	-	1.00	1.229	19.32	-
Vitvi15g01425	VIT_15s0021g01450	1.00	1.224	18.06	No hit
Vitvi13g01636	VIT_13s0158g00050	1.00	1.223	17.85	Serine carboxypeptidase
Vitvi10g01830	VIT_10s0003g02420	1.00	1.217	16.27	SRG1oxidoreductase
Vitvi17g00462	-	1.00	1.216	16.08	-

## Data Availability

RNA-seq data have been submitted in the SRA database under the accession PRJNA1132586.

## Supplementary Materials

The following supporting information can be downloaded at https://www.mdpi.com/article/10.3390/plants13182574/s1, Figure S1. Gene significance vs. module membership in selected modules; Figure S2. Optimum gene set top 10 genes; Table S1. Sequencing results read quality; Table S2. Mean normalized counts in all samples; Table S3. Differentially expressed genes in all pairwise comparisons; Table S4. Gene Set Enrichment Analysis on DEGs. Table S5. WGCNA module gene lists; Table S6. Module–genotype trait associations; Table S7. GSEA on WGCNA modules, cutoff 0.25; Table S8. GSEA on WGCNA modules cutoff 0.10; Table S9. Tau values of genes in all tissues considered in this study; Table S10. Score values of genes in all tissues considered in this study.
