# Supplementary figures and images for: The Transcriptional Landscape of Berry Skin in Red and White PIWI (“Pilzwiderstandsfähig”) Grapevines Possessing QTLs for Partial Resistance to Downy and Powdery Mildews
Source: Plants (Basel). 2024 Sep 13;13(18):2574. doi: 10.3390/plants13182574 (PMC11434962; doi:10.3390/plants13182574)

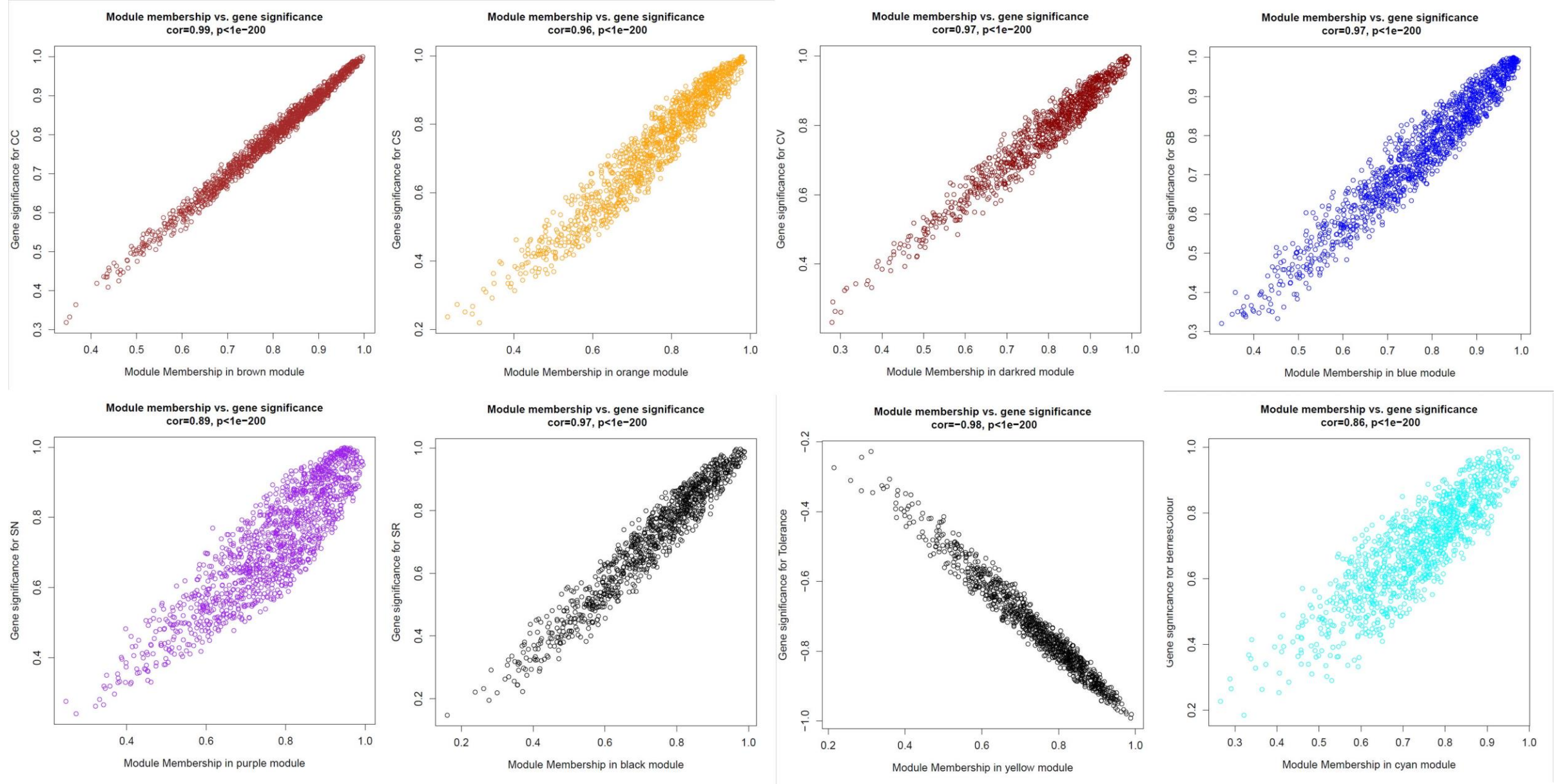

**Figure S1.** Gene Significance vs Module Membership in selected modules

Supplement: Supplementary file 1 [file plants-13-02574-s001.zip › Figure S1 - GSvsMM in selected modules.pdf]

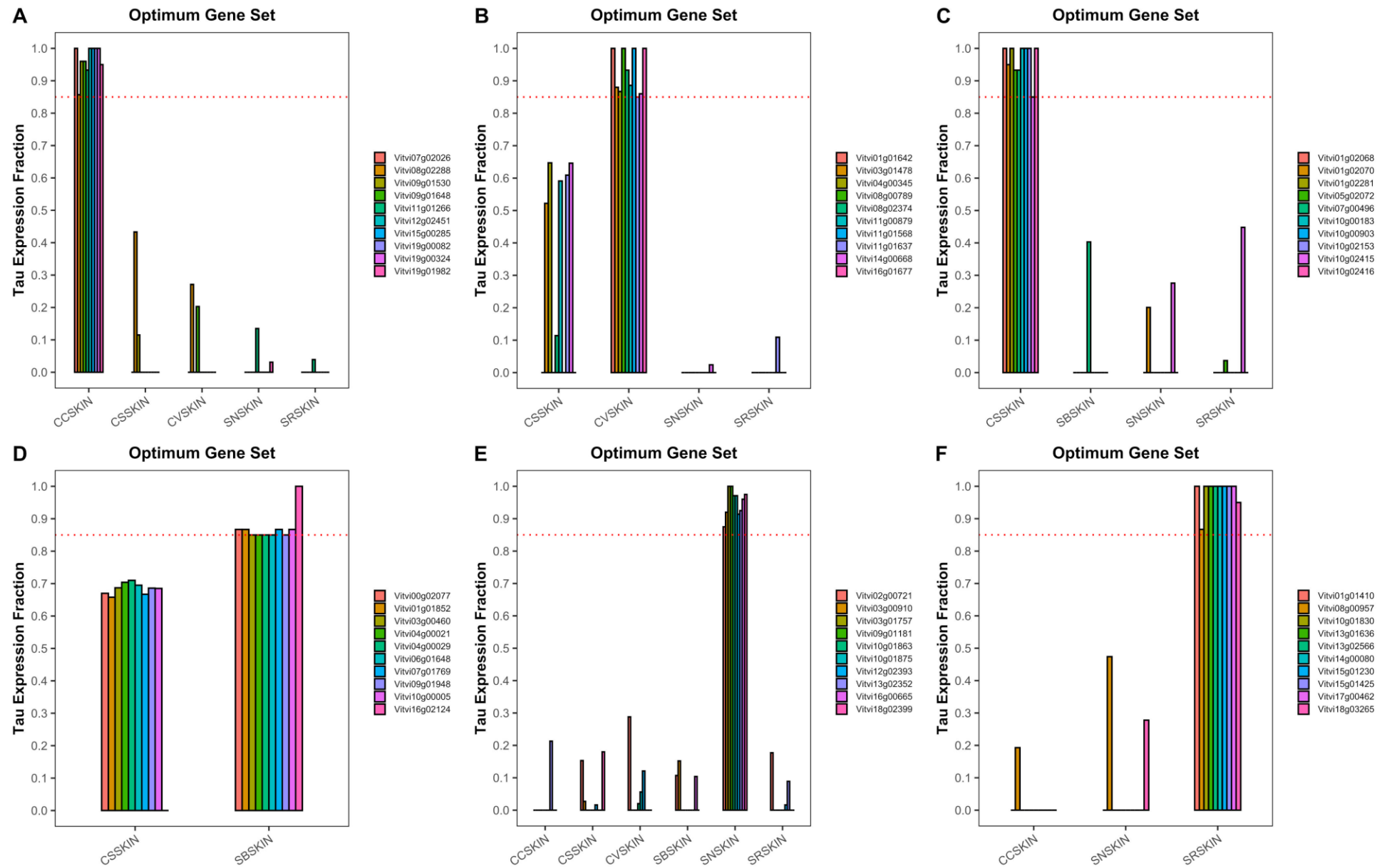

Figure S2. Optimum gene set top10 genes

Supplement: Supplementary file 1 [file plants-13-02574-s001.zip › Figure S2 - Top10 optimum genes.pdf]
